# How does psychosocial stress affect the relationship between socioeconomic disadvantage and overweight and obesity? Examining Hemmingsson’s model with data from a Danish longitudinal study

**DOI:** 10.1186/s12889-019-7699-8

**Published:** 2019-11-07

**Authors:** Per Hoegh Poulsen, Karin Biering, Trine Nøhr Winding, Ellen Aagaard Nohr, Liselotte Vogdrup Petersen, Stanley J. Ulijaszek, Johan Hviid Andersen

**Affiliations:** 1 Danish Ramazzini Centre, Department of Occupational Medicine, University Research Clinic, Regional Hospital West Jutland, Gl. Landevej 61, 7400 Herning, Denmark; 2 Institute of Clinical Research, Department of Obstetrics & Gynecology, University of Southern Denmark, Odense University Hospital, Odense, Denmark; 3 National Center for Register-Based Research, Aarhus University, Fuglesangs Allé 26, 8210 Aarhus V, Denmark; 4 Unit for Biocultural Variation and Obesity, Institute of Social and Cultural Anthropology, University of Oxford, Oxford, UK

**Keywords:** Socioeconomic disadvantage, Overweight and obesity, Psychosocial stress

## Abstract

**Background:**

Chronic stress in childhood may increase the risk of overweight and obesity in young people. Erik Hemmingsson has suggested a new obesity causation model which focuses on psychosocial stress.

The aim was to examine the associations between socioeconomic disadvantage and overweight and obesity and examine if these associations attenuate, when the effect of the different domains from Eric Hemmingsson’s obesity causation model were taken into account.

**Methods:**

A longitudinal study using data from The West Jutland Cohort Study (N = 2879). Outcome was overweight and obesity combined derived from self-reported weight and height at age 15, 18, 21 and 28 years. Exposure variables were equivalised household income, educational level and labour market participation of the mother derived from registers and psychosocial variables derived from questionnaires. A three-step adjustment model using logistic regression and stratified by gender was applied.

**Results:**

Mother’s low educational level was associated with a 3-fold increased odds of obesity in 18 year-old-girls, which attenuated when adjusting for the domains adult distress, disharmonious family environment and offspring distress. In 28 year-old girls, a 2.5-fold increased odds of obesity was observed, which attenuated when mutual adjusted for other socioeconomic variables and attenuated even further when adjusting for all the domains. In 18-year-old boys, a 3-fold increased odds of obesity was observed which attenuated after adjustments for adult distress, disharmonious family environment and offspring distress. In 21-year old boys, a four-fold increased odds of obesity was observed that attenuated after adjustments. At age 28 years, a three-fold increased odds of obesity was observed, which vanished in the fully adjusted model.

**Conclusions:**

Our study confirms to some extent that the associations between socioeconomic disadvantage and overweight and obesity can be explained by the domains included in Erik Hemmingsson’s model, although our results should be interpreted with caution. Adult distress, disharmonious family environment and offspring distress accounted for some of the association in girls, whereas in boys it was primarily offspring distress, which had the greatest impact. Young people’s educational attainment can act as a buffer in the relationship between mother’s lower educational level and obesity at age 28 years.

## Background

In western high-income countries, the prevalence of overweight and obesity has increased dramatically over the last three decades [1]. Despite a possible levelling-off among children and adolescents from more affluent families, a continued increase has been observed among lower socioeconomic classes, indicating increasing socioeconomic inequalities in overweight and obesity [2, 3]. A recent meta-analysis by Wardle et al. showed a small, yet persistent, association between perceived psychosocial stress and an increased risk of obesity in adults [4]. Among children and adolescents, overweight and obesity may have other psychosocial and social pathways than in adults. In a review by Gundersen et al., individual psychosocial stressors along with psychosocial stressors in the household were associated with an increased risk of childhood overweight and obesity [5].

The concept of stress can be defined in different ways. In the bio-physiological area, “stress” is often referred to as “the non-specific response of the body to any factors that overwhelms or threatens to overwhelm the body’s ability to maintain homeostasis” [6]. In the psychological literature, the word “stress” is often defined as “a particular relationship between the person and the environment that is appraised by the person as taxing or exceeding his or her resources and endangering his or her well-being” [7]. The experience of stress can be caused by different types of emotional challenge (e.g. unemployment or conflict) or by physiological challenges (e.g. illness) [8]. Stress can be divided into acute or chronic stress. The experience of acute stress can be related to one’s personal safety which may activate the “fight and flight” mechanism [6] and may also be associated with the inhibition of appetite/loss of appetite [9]. Chronic stress can occur in response to a prolonged exposure to psychological stressors (e.g. job pressures) as well as exposure to adverse events in childhood [10], where stress mechanisms may manifest themselves in the individual expressing a preference for high energy-dense foods [11, 12], which may contribute to weight gain and future overweight and obesity [13], especially for example among women [14].

Being obese as a child or during adolescence is a major risk factor for being obese as an adult and obesity is a major risk factor for later morbidity [15]. Obese people are often stigmatized in society which may result in severe psychological problems for the individual [16, 17]. Therefore, to shape and to help initiate future preventive initiatives against overweight and obesity in children and young people, it is important to identify psychosocial and environmental risk factors during upbringing that facilitate the experience of chronic stress in the individual.

Erik Hemmingsson recently introduced a new causal conceptual model as a possible way of rethinking preventive initiatives against obesity. The model explores the underlying reasons behind the association between low socioeconomic status (SES) and obesity with an emphasis on the psychological and emotional stress factors experienced by parents and children [18]. It is a step-by-step model of obesity causation which highlights the many steps in the lifecourse for an individual in which predisposing factors can influence the onset of weight gain. These steps are presented as domains with a wide array of psychosocial factors, where the model attempts to disentangle the possible negative effects of growing up in a socioeconomic disadvantaged environment, which eventually may lead to psychological and emotional overload in an individual and possible disrupted energy balance homeostasis, resulting in weight gain and obesity. This approach suggests that the psychosocial factors encompassed in the different domains may act as mediators for the association between socioeconomic disadvantage and obesity.

The proposed obesity causation model is primarily based on literature from the United States (US) and the United Kingdom (UK), which are countries with neoliberal political systems and high levels of inequality and insecurity at the national level, which could influence the experience of chronic stress in the population. In the US, according to the American Psychology Association, 75% of adults reported that they had experienced moderate to high levels in stress within the last month [19]. Among Americans aged 18–21 years who participated in the annual “Stress in America Survey”, 58% reported common symptoms of stress [20]. In Denmark, 40% of young women and 23% of young men aged 16–24 years reported higher levels of perceived stress according to the latest Danish National Health Profile 2017 [21] and approximately 20% of Danish children and young people aged 10–24 years reported often feeling stressed in a report, published by “The Council on Health and Disease” [22].

These reports indicate very different levels of experienced stress across countries, and perhaps stress emerges in a different way in Denmark than in the US and the UK due to a more egalitarian society with low levels of income inequality and job insecurity. The proposed step-by-step model holds promise as a new approach to understand obesity causation, and it is important to examine whether this model can be applied empirically. To examine the Erik Hemmingsson model in an empirical context, it is necessary to use longitudinal data, and to the best of our knowledge, no such examination with the use of longitudinal data has yet been performed.

Our aim was therefore to explore the associations between socioeconomic disadvantage and overweight and obesity and examine if these associations attenuate, when the effects of the domains: adult distress, disharmonious family environment, offspring distress, psychological and emotional overload and homeostasis disrupted from Eric Hemmingsson’s model were taken into account.

## Methods

### Design and population

This is a longitudinal study using data from the West Jutland Cohort Study (VestLiv), an on-going Danish study following a complete regional cohort of young people who were born in 1989 and lived in the western part of Denmark (former Ringkoebing County) in 2004. The county had a total of 275,000 inhabitants when the cohort was established in 2004.

The main purpose of this youth cohort is to study the relationship between social inequality and health from a life course perspective. The project has so far included four waves of questionnaires, in 2004, 2007, 2010 and 2017 [23], which have been supplemented with a range of register-based information. Furthermore, in 2004, the parents completed a questionnaire about the child’s health during upbringing, as well as about their own psychosocial health.

**Fig. 1:**
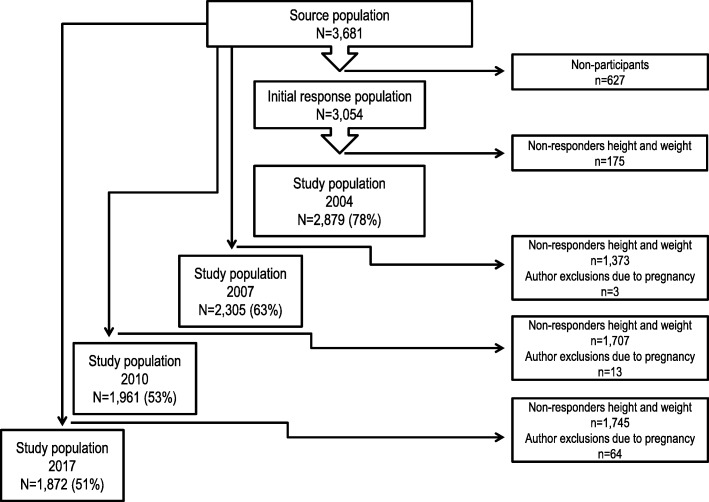
Distribution of participants and non-participants in 2004, 2007, 2010 and 2017

The source population comprised 3681 young people at the age of 15 years. Detailed information on recruitment and data collection has been described elsewhere [24]. Participants were included in this study if they had responded to questions about reported height and weight in 2004, 2007, 2010 or 2017 to determine rates of overweight and obesity. Depending on the research question, attrition and missing data reduced the sample as shown in Fig. 1. Women who were more than 3 months pregnant when they completed the questionnaire were excluded from the analyses related to this specific survey wave, due to temporally higher BMI. These exclusions are displayed in Fig. 1.

Data for this study comprised a combination of questionnaire data from both children and parents and data from registers. In Denmark, every citizen is provided with a CPR-number (Central Office of Civil Registration) at birth (or upon entry for immigrants). This is a key component for register linkages [25] and allowed us to link the CPR number of each child to parental information from registers.

### Definition of outcome

The primary outcome measure was overweight and obesity combined, defined by Body Mass Index (BMI) at age 15, 18, 21 and 28 years. Weight and height were derived from questionnaires and BMI was calculated as weight in kilograms divided by height in meters squared. At age 18–28 years, participants were categorized according to the International Classification of adult obesity (BMI ≥ 30 kg/m^2^) [26, 27]. However, at age 15 years, participants were categorized into “normal weight” (< 23.29 kg/m^2^ for boys and < 23.94 kg/m^2^ for girls), and “overweight” (BMI ≥23.29 kg/m^2^ for boys and BMI ≥23.94 kg/m^2^ for girls) using thresholds for 15 year old girls and boys [28] because of few obese at this age (21 girls and 23 boys).

### Definition of exposure domains

**Fig. 2:**
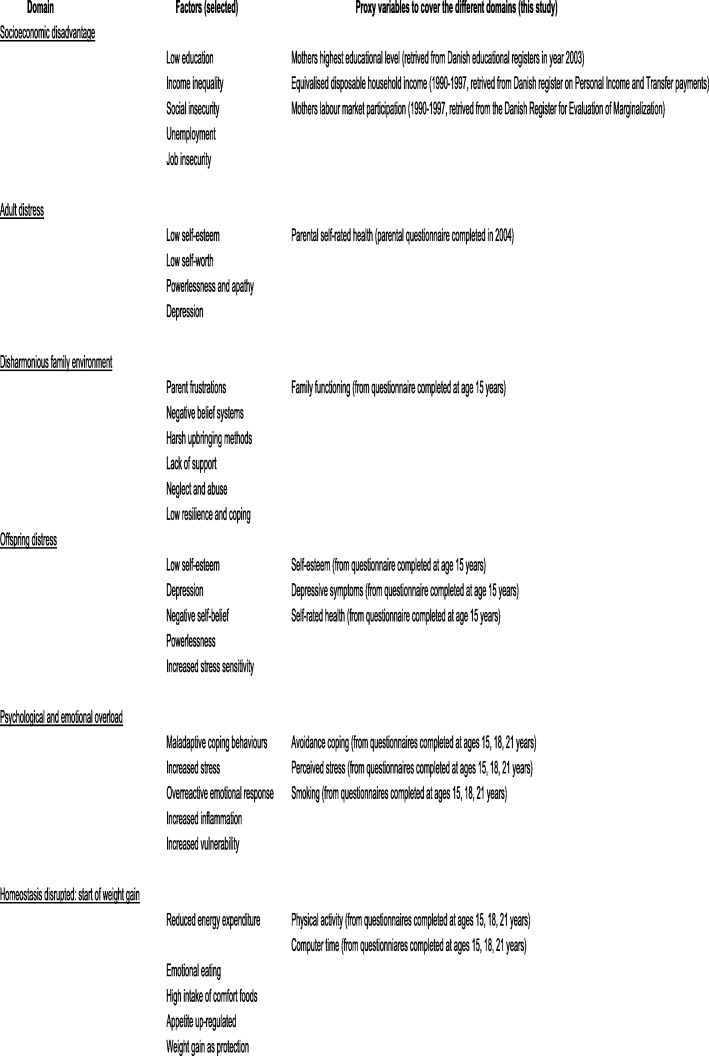
presents the domains from Hemmingsson’s model with the chosen proxy variables in this study (adapted from Fig. 1, Hemmingsson 2014)

We generated proxy variables from registers and questionnaires for the six domains in Hemmingsson’s causation model: socioeconomic disadvantage, adult distress, disharmonious family environment, offspring distress, psychological and emotional overload, and homeostasis disrupted: start of weight gain (hereafter referred to as homeostasis disrupted). These domains are adapted from Fig. 1 in [18], presented in Fig. 2, and explained in detail below.

*Socioeconomic disadvantage* was measured as mother’s highest educational level, equivalised disposable household income and mother’s labour market participation. Information on mother’s highest educational level in 2003 was derived from different educational registers [29]. The variable was divided into three categories: ≤10 years (primary school), 11–13 years (secondary school) and > 13 years of education (tertiary school). If information was missing for year 2003, information from previous years was applied (last observation carried forward).

Annual equivalised disposable household income (equivalised income) was included as it informs about the inequality in wealth distribution among Danish families independent of family size and age distribution in the family. Equivalent disposable income is a weighted value which uses an equivalence scale that takes into account that a family of two adults consumes more, but does not need twice the income as a family with only one adult. The scale also reflects that children do not need as much income as adults to achieve the same standard of living. Information about equivalised income in Danish Kronor (DKK) was derived from the Danish register on Personal Income and Transfer Payments [30] and we applied information from 1990 to 1997 (8 years). We calculated a mean value for this early childhood period and categorized the variable into low, medium and high equivalised income, grouped by the 33.3rd and 66.6th percentiles. Information on mother’s labour market participation (LMP) was derived from the Danish Register for Evaluation of Marginalization (referred to as the DREAM Register) which provides information on public transfer incomes on a weekly basis [31]. Mother’s LMP was defined according to the degree of receiving social benefits (e.g. sickness absence compensation or unemployment benefits) within each year from the second half of 1991 to 1997. When we defined this variable, we omitted maternity leave benefits or state educational grants. LMP was a continuous variable in the range from 0 to 100 and calculated as a mean LMP score between 0 and 1 for the entire period and categorized into “high LMP” and “low LMP” at a cut-off value of ≥0.80 indicating high LMP.

*Adult distress* was measured as parental self-rated health (2004). Information was provided by the parents in the parental questionnaire in 2004 and measured using a single item from the SF-36 on general health (GH-1) [32]. The question was: “In general, would you say your health is …” with five response options ranging from “excellent” to “poor”, which was subsequently dichotomised to indicate “good” (excellent, very good) versus “poor” (good/less good/poor) self-rated health.

*Disharmonious family environment* was measured as family functioning. Information on family functioning came from the baseline questionnaire in 2004, when the participants were 15 years of age. Family functioning was a categorical variable based on the general functioning subscale of the McMaster Family Assessment Device (FAD), developed by Epstein et al. [33]. The FAD consists of seven subscales where General Functioning assesses the overall health/pathology of the family with questions about how the family handles such things as crisis and other family issues. It consists of 12 items with four response categories ranging from “strongly agree” to “strongly disagree” (scores of 1–4), where higher values indicate poorer family functioning. We calculated a mean value for the 12 items. A pragmatic decision was made by the authors to include participants with 8 and more answers to enhance the number of participants, despite missing items. The variable was dichotomised at the 75th percentile of the mean value indicating poor family functioning at ≥2.08, which lies between the mean value for the non-clinical and clinical samples on General Functioning [33]. This cut-off value has been applied in previous studies on the same cohort.

*Offspring distress* was measured as participant’s self-rated health, self-esteem and depressive symptoms. From the baseline questionnaire, we used information about self-rated health, self-esteem and depressive symptoms.

Self-rated health was measured using a single item from SF-36 on general health (GH-1) and the response categories were dichotomised into two groups: “good” self-rated health (excellent/very good), and“poor” self-rated health (good/less good/poor) as described above with the domain adult distress [32]. Self-esteem was measured using six items from the Rosenberg self-esteem scale with scores from 1 to 4 and a total score between 6 and 24 [34]. Scores were reversed so higher scores indicated lower self-esteem. The variable was dichotomised at the 75th percentile into “high” and “low” self-esteem. Depressive symptoms were measured using the abbreviated 4-item validated version of “The Center for Epidemiologic Studies Depression Scale for Children” (CES-DC) [35]. It consists of four items asking about one’s mental state over the past week. There are four categories of answers to each question ranging from “not at all” to “a lot”. The answers are awarded scores of 0–3, where high values correspond to having depressive symptoms. We applied single item imputation if one item was missing for the scale by adding the mean of the other items. The four items summed up to a score between 0 and 12. The definition of depressive symptoms was obtained by using the cut-off point of 3 and above indicating depressive symptoms as recommended for the short scale by Fendrich et al. [35].

*Psychological and emotional overload* was measured as avoidance coping, perceived stress and smoking status. Information about avoidance coping, perceived stress and smoking status was collected from the 2004, 2007 and 2010 questionnaires. Avoidance coping was measured using three subscales of two items each from the BRIEF COPE Scale [36]. The three subscales employed in this study were “self-distraction”, “substance use” and “behavioural disengagement”. Each item had 4 response categories yielding scores between 1 and 4, with higher scores indicating a higher level of avoidance coping. The avoidance coping scale was created by the mean of the item scores. The distribution of avoidance coping for this population was skewed to the right, so we decided to dichotomise the avoidance coping scale into low and high avoidance coping at the 75th percentile, respectively.

Perceived stress was measured using a Danish 4 item version of the Perceived Stress Scale (PSS), which was originally developed by Cohen et al. [37]. The 4 items ask about the responder’s experience of being in control of their life during the last month. Each item has a score of between 0 (“never”) and 4 (“very often”). The total scale ranged from 0 to 16 points where higher values indicated higher levels of perceived stress. PSS has no clinical cut points, so the variable was dichotomised into low and high PSS at the 75th percentile, respectively.

Smoking status was a categorical variable with four possible answers that were dichotomised into smoking (“yes, but not every week”, “yes, but not every day”, “yes, daily”) and not smoking (“no, I do not smoke”).

*Homeostasis disrupted* was measured as physical activity and computer time. Information about physical activity and computer time was collected from questionnaires in 2004, 2007 and 2010.

Physical activity (PA) was a categorical variable with six possible answers where each participant was asked in a single item, “How many hours a week during leisure time do you usually exercise or play sports where you are out of breath or sweating?”. The answer categories of PA were respectively: none, ½ hour, 1 h, 2–3 h, 4–6 h, and 7 h or more. The variable was dichotomised according to the recommendation on PA given by the Danish Health Authorities for adolescents (60 min/day) and young adults (30 min/day) [38]. At age 15 years, the variable was dichotomised into: “Low level of PA” (≤2–3 h/week); “high level of PA” (≥4–6 h/week) assuming 2 h of compulsory physical education classes at school. At age 18 and 21 years, the variable was dichotomised into: “Low level of PA” (≤1 h/week); “high level of PA” (≥2–3 h/week).

Computer time (CT) was a categorical variable with 7 possible answers where each participant was asked in a single item, “On an average (school) day, how many hours of your leisure time do you spend in front of a computer?”. The answer categories of CT were in the range of “I am not using the computer” to “Approximately five hours or more per day”. Since we do not have any official Danish recommendations for children’s and young people’s computer use, the authors made a pragmatic decision to dichotomise the variable at the 75th percentile, which resulted in slightly different cut-offs. At age 15, the variable was categorised into “low level of CT” (≤2 h/day) and “high level of CT” (≥3 h/day). At age 18 and 21 the variable was categorised into “low level of CT” (≤3 h/day) and “high level of CT” (≥4 h/day).

### Additional variables

Birth-weight and highest educational level at age 28 years.

Birth-weight was included in the analyses because high birth-weight has previously been associated with later overweight and obesity [39]. Information on birth-weight was obtained from the Danish Medical Birth Register, which is a national register with information about all hospital and home births [40].

As a proxy for the participant’s own socioeconomic position at age 28 years, we obtained information on highest educational level from educational registers [29]. The variable was divided into three categories: ≤10 years, 11–13 years and > 13 years of education.

### Statistical analyses

We calculated proportions on each variable from the six domains in relation to the outcome at age 15–28 years, stratified by gender. Logistic regression models were used to calculate the associations between each of the three main exposures (socioeconomic disadvantage domain) and overweight and obesity at age 15–28 years. Estimates are presented as odds ratios (OR) with 95% confidence intervals (95% CI). We also examined each of the variables from the remaining 5 domains individually with the outcome at age 15–28 years using logistic regression.

In the main regression analyses it was decided á priori to include variables from the other domains as potential confounders in a three-step adjustment model. We examined the correlations between variables within each domain using Spearman’s Rank correlation coefficient to ensure that we did not apply highly correlated variables from the same domains to the models, which could increase the risk of over-adjustments. Self-esteem, depressive symptoms and self-rated health (offspring distress) were correlated with Spearman’s rho = 0.28 and 0.37. Perceived stress and avoidance coping (psychological and emotional overload) were correlated with Spearman’s rho = 0.40. The rest of the correlations between proxies within domains were similar or smaller (correlation matrix not shown).

In the first model (crude), we examined the association between each of the three socioeconomic variables (socioeconomic disadvantage) and overweight and obesity at age 15–28 years (Model I). In the second model, we mutually adjusted for the other SES variables, because we wanted to examine the independent effect of each SES variable in relation to overweight and obesity (Model II). In the third model (Model III), we adjusted for Model II variables and the domains: *adult distress*, *disharmonious family environment* and *offspring distress*. In the fourth and fully adjusted model (Model IV), we adjusted for Model II + Model III and the domains: *psychological and emotional overload* and *homeostasis disrupted*. We included the exposure variables for the two domains *psychological and emotional overload* and *homeostasis disrupted* at age 15, 18 and 21 years ensuring that exposures were measured before the outcome at age 18–28 years. Thus, when we examined the outcome at age 18 years, the exposures were measured at age 15 years.

At age 28 years, we also included an adjustment for the young people’s highest educational level. Additionally, we adjusted for birth-weight as a continuous variable in model III-IV at all four time-points.

We assumed that there was no interaction between the variables from the socioeconomic disadvantage domain and the proxy variables from the other domains.

We explored the adjusted effect of the individual proxies in the association between the socioeconomic disadvantage domain and the outcome at all four time-points in supplementary analyses (tables not shown).

All analyses were stratified by gender.

Data-analysis was performed by the statistical package Stata, statistical software version 14.2 (Stata Corporation, College Station, Texas, USA).

### Ethics

Use of the data was carried out under the same conditions and with the same purpose as when originally collected and based on approval from the Danish Data Protection Agency and their rules for data protection. According to Danish law at the time of data collection, approval by the Ethics Committee and written informed consent were not required for questionnaire-based and register-based projects.

## Results

Tables 1 and 2 present the proportion of overweight and obese girls and boys at age 15, 18, 21 and 28 years in relation to the proxies in each domain.

**Table 1:**
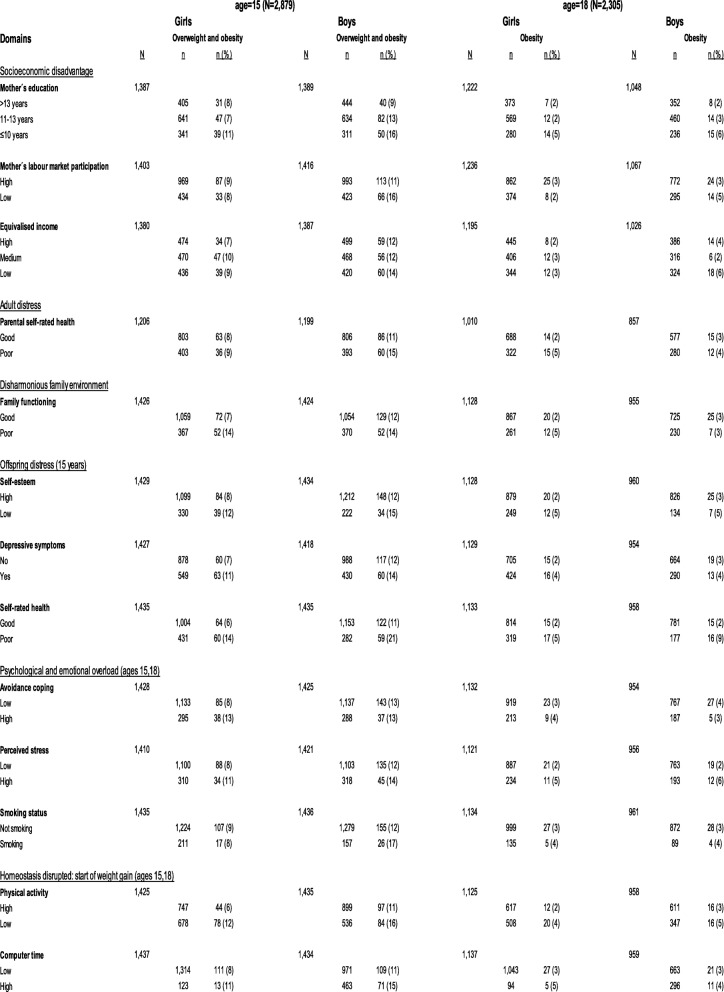
Distribution of proxy variables from the domains (Hemmingsson:2014) in relation to the outcome at ages 15 and 18, stratified by gender

**Table 2:**
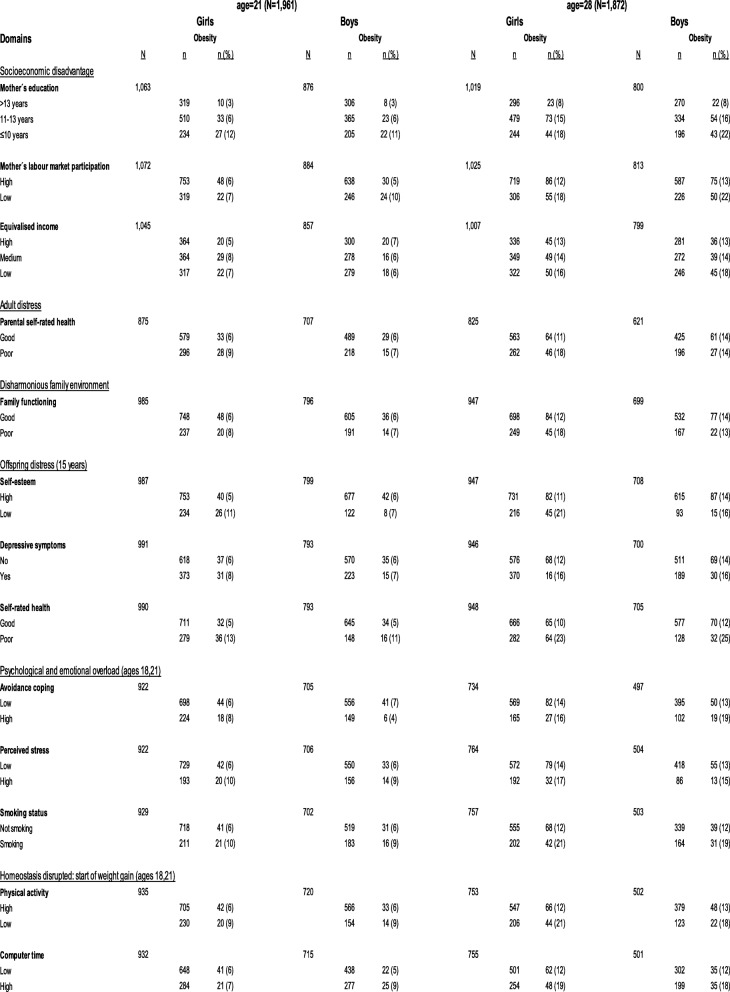
Distribution of proxy variables from the domains (Hemmingsson:2014) in relation to the outcome at ages 21 and 28, stratified by gender

A higher proportion of overweight and obese girls were observed at ages 15, 18, 21 and 28 years if they grew up having a mother with a low level of education. At all four time points, a higher proportion of overweight and obese girls were also observed if they reported poor family functioning, lower self-esteem, poor self-rated health, high avoidance coping, perceived stress, low level of PA, or a high amount of CT. Furthermore, a higher proportion of obese girls at ages 21 and 28 years were smokers.

A higher proportion of overweight and obese boys were observed at age 15, 18, 21 and 28 years if they grew up having a mother with a low level of education or their mothers had a low labour market participation. A higher proportion of overweight and obese boys was also observed at all 4 time points if they reported poor self-rated health, higher levels of perceived stress, were a smoker, had low level of PA or a high amount of CT.

Additional file 1: Table S1 and Additional file 2: Table S2 present the crude estimates for the assocation between proxy variables for the domains *adult distress* to *homeostasis disrupted* and overweight and obesity at age 15–28 years in girls and boys, respectively.

Parental poor self-rated health (adult distress) was associated with overweight and obesity at age 18–28 years in girls and at age 15–18 years in boys. Poor family functioning (disharmonious family environment) was associated with overweight and obesity at age 15, 18 and 28 years in girls, but not boys. Low self-esteem, depressive symptoms and poor self-rated health (offspring distress) were associated with overweight and obesity at age 15–28 years in girls, in boys merely poor self-rated health was associated with overweight and obesity at age 15–28 years. High avoidance coping, perceived stress and smoking (psychological and emotional overload) among girls were associated with overweight and obesity at ages 15–18, 18–21 and 21–28 years, respectively. High perceived stress was in boys primarily associated with obesity at age 18 years, where smoking showed increased odds of obesity at age 28 years. In girls, PA (homeostasis disrupted) was associated with overweight and obesity at all four time-points, where CT was associated with obesity at age 28 years. In boys, this picture was similar to the girls for the domain.

### Socioeconomic disadvantage and overweight and obesity (Table 3, girls)

When we examined the association between mother’s educational level and overweight and obesity in 15 year-old girls, our results only revealed a tendency towards an association which was not influenced by any adjustment. At age 18 years, an almost three-fold increased odds for obesity was observed among girls with lower educated mothers, compared to girls with higher educated mothers. This attenuated primarily in Model III, whereas further adjustment in Model IV did not alter the estimates. At age 21 years, odds of obesity were four-fold greater, and this was not influenced by income or mother’s LMP (Model II). When we included the variables from the domains in Model III, the estimates increased and showed a more than 5-fold increased odds for obesity, which did not change in the fully adjusted model. At age 28 years, we observed a more than 2.5-fold increased odds for obesity in girls with lower or medium educated mothers, which attenuated slightly by adding equivalised income and mother’s LMP in Model II. When we included variables from the domains in Model III estimates increased slightly. Adding further to the model in terms of variables included in Model IV attenuated the association considerably in girls with lower educated mothers.

**Table 3:**
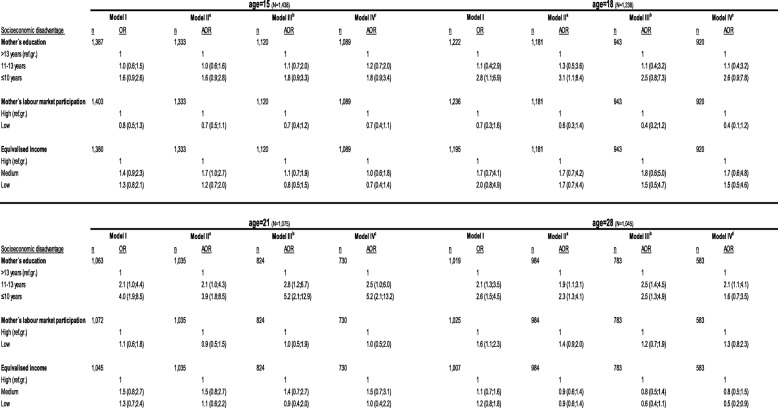
Unadjusted and adjusted estimates for the association between the socioeconomic disadvantage domain and overweight and obesity at age 15, 18, 21 and 28 years (girls)

a Mutual adjustments (adj.) for other SES variables

b Adj. for Model II + adult distress, disharmonious family environment and offspring distress, birth-weight

c Adj. for Model II + III, psychological and emotional overload, homeostasis disrupted, birth-weight

d Adj. for Model II + III, psychological and emotional overload, homeostasis disrupted, birth-weight, young people’s own education (age 28)

We did not find consistent associations between mother’s low LMP and overweight and obesity at age 15, 18 and 21 years. At age 28 years there was 1.6-fold increased odds of obesity, which attenuated by adding variables included in Model II + III. Estimates did not change in the fully adjusted model. When we examined the associations between low equivalised income and overweight and obesity at ages 15 to 28 years, the majority of the associations showed small and inconsistent results. However, at age 18 years results showed 2-fold increased odds for obesity, which attenuated when adding variables included in Model II + III. The fully adjusted model did not change the estimates.

### Socioeconomic disadvantage and overweight and obesity (Table 4, boys)

When we examined the association between mother’s educational level and overweight and obesity in 15 year-old boys, we observed a 1.9-fold increased odds of overweight and obesity in boys with lower educated mothers, compared to boys with higher educated mothers. Estimates did not change much when we added equivalised income and mother’s LMP to the second model, and adding variables in Model III + IV did not reveal further changes. Among 18-year-old boys, we observed a 3-fold increased odds of obesity, which attenuated with the inclusion of equivalised income and mother’s LMP in the second model. Adding the domains adult distress, disharmonious family environment and offspring distress to the third model attenuated the associations even further. In the fully adjusted Model IV, the estimate increased slightly. At age 21 years, we observed a more than four-fold increased odds for obesity in boys having a mother with low level of education and it was primarily by adding equivalised income and mother’s LMP to the second model that attenuated the associations. When applying the fully adjusted model, estimates attenuated slightly more. At age 28 years, we observed a more than 3-fold increased odds for obesity, the associations being primarily attenuated in Model III by adding equivalised income, mother’s LMP and variables from the domains adult distress, disharmonious family environment and offspring distress. When we applied the fully adjusted model the association between mother’s low educational level and obesity vanished.

**Table 4:**
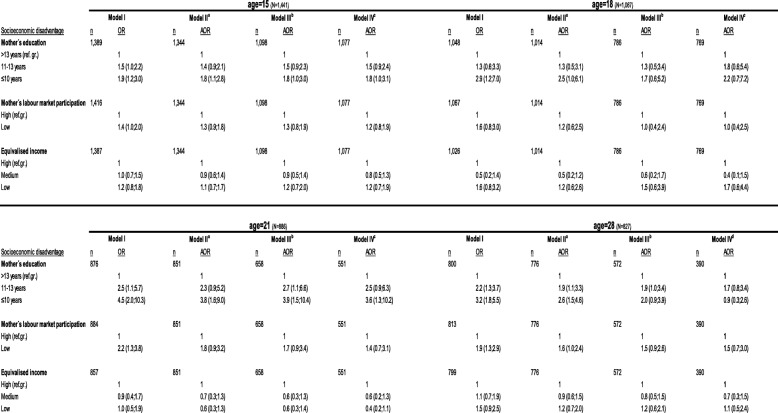
Unadjusted and adjusted estimates for the association between the socioeconomic disadvantage domain and overweight and obesity at age 15, 18, 21 and 28 years (boys)

a Mutual adjustments (adj.) for other SES variables

b Adj. for Model II + adult distress, disharmonious family environment and offspring distress, birth-weight

c Adj. for Model II + III, psychological and emotional overload, homeostasis disrupted, birth-weight

d Adj. for Model II + III, psychological and emotional overload, homeostasis disrupted, birth-weight, young people’s own education (age 28)

When we examined the associations between mother’s low LMP and overweight and obesity in boys it appeared that the association at age 15 years to some extent attenuated when all variables from the different domains were included in the fully adjusted model. This tendency was also seen at age 21 years. At ages 18 and 28 years it was primarily by adding mother’s LMP, equivalised income and the variables from the domains adult distress, disharmonious family environment and offspring distress which attenuated the associations.

## Discussion

The aim of this study was to explore the associations between socioeconomic disadvantage and overweight and obesity and to examine if these associations attenuate, when the different domains from Eric Hemmingsson’s obesity causation model were taken into account. Our results showed that mother’s lower educational level as proxy for the socioeconomic disadvantage domain was by far the strongest and most consistent risk factor for overweight and obesity at ages 15 to 28 years in both genders with an up to 4-fold increased odds for overweight and obesity. Mother’s low LMP was a consistent risk factor in relation to overweight and obesity in boys only.

For both genders, controlling for the different domains when examining the association between mother’s low educational level and overweight and obesity did not influence the associations much at age 15 years, while at age 21 years, some gender-differences became apparent. In the analysis with the outcome in 18-year-old girls and boys it appeared that adjusting for especially the variables included in the domains adult distress, disharmonious family environment and offspring distress attenuated the associations to some degree. At age 21 years, however, adjustments increased the association in girls, whereas in boys the association attenuated. At age 28 years the estimates attenuated considerably in both genders when we added all the variables in the fully adjusted model. For both girls and especially boys it appeared that the introduction of their own educational level in the models substantially decreased the ORs for the association between mother’s low educational level and obesity, which points to a potential strong buffering effect of education for the development of overweight and obesity in adulthood [41].

Our study showed that the associations between socioeconomic disadvantage and overweight and obesity to some degree attenuated when the domains from Erik Hemmingsson’s obesity causation model were taken into account. This may, to some extent, confirm that the proposed obesity causation model can be used as a model to understand overweight and obesity among young people living in a more egalitarian society. Our choice of proxies for the different domains may, however, have influenced our findings and makes it difficult to examine the model in full; this will be discussed in further details under analytic approach and limitations.

When we examined the associations between low equivalised income and overweight and obesity we found no association. The Danish well-fare society is well organized and individuals have the opportunity of receiving social benefits in case of long-term unemployment or sick leave which may, to some degree, decrease the risk of chronic stress related to financial difficulties compared to the US and the UK. Danish parents have furthermore the opportunity to stay on parental leave for a longer period than in most other countries, which perhaps decreases the risk of parental distress experienced during this stressful period of starting up a family. Our data showed that boys who grew up in families with a mother having a low LMP in early childhood had some increased odds of overweight and obesity. In girls, the tendency was opposite, and we have no good explanation for this difference. The results in boys may reflect that mother’s with low LMP or being unemployed in early childhood have less surplus to, for example, prepare healthy nutritious food which along with increased sedentary behaviour, unhealthy eating habits [42] and disturbed sleep pattern [43] in adolescent boys may increase the risk of overweight and obesity.

Our analyses showed that mother’s low educational level as a proxy for the socioeconomic disadvantage domain was the most stable and consistent risk factor for overweight and obesity in both genders. This may add attention to the different forms of social and cultural capital [44] which may be passed on from parent’s to children, due to the fact that children from families of lower socioeconomic status may carry much less capital compared to peers from families of higher socioeconomic status [45]. In this Danish context, cultural capital may be very important, since children who grow up in families with parents having a low level of education more often end up with a lower educational attainment [46], which may increase risks of unhealthy habits related to lifestyle and health. Our results revealed a quite strong role of own education in mitigating the relationships between maternal lower educational level and young people’s obesity at age 28 years. It seems therefore essential to address the importance of young people’s educational attainment since this, at least to some extent, may prevent overweight and obesity.

Our analyses showed that the associations between mother’s low educational level and obesity at age 18 years attenuated primarily when we added the variables from the domains adult distress, disharmonious family environment and offspring distress. Self-rated health of the participants (offspring distress) was a robust and consistent risk factor for overweight and obesity at all four ages in both genders, which could indicate that this variable may account for some of the effect. This is supported by the findings from supplementary analyses (results not shown) where we did adjustments for the individual proxies which showed that participant’s poor self-rated health attenuated the associations substantially, especially in boys. In girls, however, the variables parental poor self-rated health (adult distress) and poor family functioning (disharmonious family environment) also attenuated the associations to some degree.

It is important to address the fact that every fifth child or young person aged between 10 and 24 years reported often feeling stressed [22] and further disentangle whether this is related to family conflict, well-being in schools or increased job demands, which may have the potential of evolving to chronic stress with negative health consequences.

Our results have shown that especially mother’s lower educational level was associated with later overweight and obesity in both genders. It is therefore important to increase the support to socioeconomically disadvantaged families during childhood to help decrease stress in parents which may influence the family environment where the child is living. Likewise it is important to address the attention to children and young people’s report of poor self-rated health since this may act as an important marker of later overweight and obesity. It therefore also seems relevant to include a greater use of self-report from children and adolescents due to its value to get more good surveillance data to be able to better target preventive initiatives within overweight and obesity.

Obesity in children and young people is a very complex issue which makes it difficult to be specific in relation to preventive initiatives. However, being stressed due to e.g. poorer family function or/ and having a poor self-rated health as a child or adolescent may increase the risk of applying maladaptive coping mechanisms and induce risky behaviours which may track into adulthood and increase the risk of poorer health later on. It is therefore important to address these issues at the family and school level since they appear to be important steps on the pathway between socioeconomic disadvantage and obesity, at least in a Danish context.

### Analytic approach

We applied proxy variables for all the domains which were available from surveys and registers. It may be debatable whether these proxies were sufficient and robust enough to capture the content of the domains presented in Erik Hemmingsson’s model and perhaps less suited to be applied to both genders. As presented under the results several of the proxies appeared to pertain primarily to girls which may have influenced our results.

The overall avoidance scale included items about substance use. Previous studies have found an association between maladaptive coping mechanisms and obesity [47]. It can be speculated that if a person applied this type of maladaptive coping mechanism for chronic stress, perhaps overeating as well could be implied in this kind of substance use, especially among girls [48].

We did not have the opportunity to include information about more severe childhood adversities such as parental neglect in childhood, which has shown to be an important risk factor for later obesity [49], nor about childhood abuse [50], which may severely increase psychosocial distress in children. Including information on parental divorce or single-mother background could be relevant since being a single mother may increase distress which can potentially influence the family environment and induce increased psychosocial distress in children and hence lead to an increase in weight [51].

Our analytic approach was a three-step model with adjustments for the proxy variables in the different domains as potential confounders. Since some of the proxy variables within offspring distress and within psychological and emotional overload to some extent were correlated we did a supplementary analysis for both genders, where we only included the overall strongest proxies in each domain in relation to the outcome. This did, however, not change the estimates radically (results not shown).

We included adjustments for birth weight in Model III + IV, and it is debatable whether the attenuation of the estimates may be due to this adjustment or to the included variables in the different domains. We did supplementary analyses between the different exposures and the outcome, adjusting solely for birth weight; this did, however, not change the estimates much, so the attenuation of estimates is likely due to the other included variables and not birth weight (results not shown).

#### Strengths and limitations

To our knowledge, this is the first study to examine this Hemmingsson obesity causation model using longitudinal data to disentangle the associations between socioeconomic disadvantage during childhood and overweight and obesity in adolescence and early adulthood.

A major strength of this study was the fact that it was a prospective cohort study using data from four survey waves in the West Jutland Cohort Study, supplemented with register information on the three socioeconomic exposure variables, resulting in few missing values on the main exposures.

One of the main limitations of the study was that the main outcome was based on self-reported weight and height and several of the applied proxies was also based on self-reported information, which is prone to misclassification. Participants in surveys, who are overweight or obese, are probably more likely to underestimate weight, especially girls [52] which may increase the risk of differential misclassification. This increases the risk of overestimating a potential association and hence bias away from the null hypothesis. We acknowledge the fact that the measured associations at age 15 years were cross-sectional and cannot tell us anything about the direction of associations. We did not find the model suitable to explain the associations between mother’s low educational level and overweight and obesity at age 15 years. This may, however, be attributed to the fact that we applied the BMI limits for overweight and not obesity due to very few obese subjects at this age. As mentioned in the section about the analytic approach our chosen proxies may not fully cover the different domains in Hemmingsson’s model which limits the ability to examine the model in full. However, we have included available variables which we believe may act as proxies for the different domains. Unfortunately, we did not have information on food intake for the domain regarding homeostasis disrupted which may have influenced our results. It may also be debatable whether applying smoking status as a proxy for the psychological and emotional overload domain seems reasonable, however, we believe that smoking may reflect a maladaptive coping mechanism which was not covered by the questions regarding substance use.

The cut-off for high level of PA in adults was set below the recommended limit for weekly PA, which is due to the response categories and also to ensure that we did not get any rendered results because there were quite few 21 year olds having a PA level of ≥4 h per week.

We chose to dichotomise many of the continuous and categorical proxy variables to facilitate comprehensibility of the results although dichotomising a variable will result in loss of information [53].

A previous examination of the study setting concluded that the participants of this youth cohort do not differ from young people in other parts of Denmark [54]. The results from this study with the abovementioned limitations may therefore be generalizable to other young people experiencing environmental and social conditions similar to this Danish youth cohort.

## Conclusion

Our study confirms to some extent that the associations between socioeconomic disadvantage and overweight and obesity can be disentangled by the domains included in Erik Hemmingsson’s proposed obesity causation model. Our results showed that mother’s low educational level as a proxy for socioeconomic disadvantage was clearly associated with overweight and obesity in both gender with an up to four-fold increased odds, whereas mother’s low LMP was associated with overweight and obesity in boys only. Poor parental self-rated health (adult distress), poor family function (disharmonious family environment) and poor self-rated health (offspring distress) of the participant’s appeared to account for some of the effect in girls, in boys this was merely poor self-rated health (offspring distress). Young people’s own educational attainment may act as a buffer of the association between mother’s low educational level and obesity at age 28. The main results should be interpreted with caution due to the risk of information bias related to the outcome and due to the fact that some of the chosen proxies for the different domains may pertain primarily to girls and may not fully cover the domains of Hemmingsson’s model.

Future research should focus on other proxy variables which may pertain to earlier stages in childhood to further explain the associations between socioeconomic disadvantage and overweight and obesity in the offspring and to further investigate whether the gender differences found in our study may be due to the chosen proxies or the included ages of outcome. It seems important to include information about e.g. parental neglect and childhood abuse in future studies because of their strong associations with later obesity. To prevent overweight and obesity in children and young people, it is important that societies address the experience of stress among especially socioeconomic disadvantaged families. It also seems essential to address the importance of young people’s educational attainment given the potential important mitigating role of own education in the relationship between maternal low education and later overweight and obesity.

## Supplementary information

**Additional file 1: Table S1.** Unadjusted estimates for the association between the individual proxies from the 5 domains (Hemmingsson:2014) and overweight and obesity at age 15, 18, 21 and 28 years (girls).

**Additional file 2:** **Table S2.** Unadjusted estimates for the association between the individual proxies from the 5 domains (Hemmingsson:2014) and overweight and obesity at age 15, 18, 21 and 28 years (boys).

## Data Availability

The data that support the findings of this study are available from Statistics Denmark but restrictions apply to the availability of these data, which were used under license for the current study, and so are not publicly available. Data are however available from the authors upon reasonable request and with permission of Statistics Denmark.

## Abbreviations

AOR: Adjusted Odds Ratio
BMI: Body Mass Index
CES-DC: Center for Epidemiologic Studies Depression Scale for Children
CI: Confidence interval
CT: Computer time
E.g.: Exempli gratia
FAD: McMaster Family Assessment Device
LMP: Labour market participation
OR: Odds ratio
PA: Physical activity
PSS: Perceived Stress Scale
SES: Socioeconomic status
UK: United Kingdom
US: United States
