# Supplementary material for: How does psychosocial stress affect the relationship between socioeconomic disadvantage and overweight and obesity? Examining Hemmingsson’s model with data from a Danish longitudinal study
Source: BMC Public Health. 2019 Nov 7;19:1475. doi: 10.1186/s12889-019-7699-8 (PMC6839153; doi:10.1186/s12889-019-7699-8)
Supplement: Supplementary file 1 — Additional file 1: Table S1. Unadjusted estimates for the association between the individual proxies from the 5 domains (Hemmingsson:2014) and overweight and obesity at age 15, 18, 21 and 28 years (girls). [file 12889_2019_7699_MOESM1_ESM.pdf]

Table S1. Unadjusted estimates for the association between the individual proxies from the 5 domains (Hemmingsson:2014) and overweight and obesity at age 15, 18, 21 and 28 years (girls).

	**age=15** (N=1,438)		**age=18** (N=1,238)		**age=21** (N=1,075)		**age=28** (N=1,045)	
Adult distress								
**Parental self-rated health**	1,206		1,010		875		825	
Good		1		1		1		1
Poor		1.2 (0.8;1.8)		2.4 (1.1;4.9)		1.7 (1.0;2.9)		1.7 (1.1;2.5)
Disharmonious family environment								
**Family functioning**	1,426		1,128		985		947	
Good		1		1		1		1
Poor		2.3 (1.5;3.3)		2.0 (0.9;4.2)		1.3 (0.8;2.3)		1.6 (1.1;2.4)
Offspring distress (15 years)								
**Self-esteem**	1,429		1,128		987		947	
High		1		1		1		1
Low		1.6 (1.1;2.4)		2.2 (1.0;4.5)		2.2 (1.3;3.7)		2.1 (1.4;3.1)
**Depressive symptoms**	1,427		1,129		991		946	
No		1		1		1		1
Yes		1.8 (1.2;2.6)		1.8 (0.9;3.7)		1.4 (0.9;2.3)		1.5 (1.0;2.1)
**Self-rated health**	1,435		1,133		990		948	
Good		1		1		1		1
Poor		2.4 (1.6;3.4)		3.0 (1.5;6.1)		3.1 (1.9;5.2)		2.7 (1.9;4.0)
Psychological and emotional overload (ages 15,18,21)								
**Avoidance coping**	1,428		1,132		922		734	
Low levels		1		1		1		1
High levels		1.8 (1.2;2.7)		1.7 (0.8;3.8)		1.3 (0.7;2.3)		1.2 (0.7;1.9)
**Perceived stress**	1,410		1,121		922		764	
Low levels		1		1		1		1
High levels		1.4 (0.9;2.2)		2.0 (0.9;4.3)		1.9 (1.1;3.3)		1.2 (0.8;2.0)
**Smoking status**	1,435		1,134		929		757	
Not smoking		1		1		1		1
Smoking		0.9 (0.5;1.6)		1.4 (0.5;3.7)		1.8 (1.1;3.2)		1.9 (1.2;2.9)
Homeostasis disrupted (ages 15,18,21)								
**Physical activity**	1,425		1,125		935		753	
High levels		1		1		1		1
Low levels		2.1 (1.4;3.1)		2.1 (1.0;4.3)		1.5 (0.9;2.6)		2.0 (1.3;3.0)
**Computer time**	1,437		1,137		932		755	
Low		1		1		1		1
High		1.3 (0.7;2.3)		2.1 (0.8;5.6)		1.2 (0.7;2.0)		1.6 (1.1;2.5)
