# Supplementary material for: How does psychosocial stress affect the relationship between socioeconomic disadvantage and overweight and obesity? Examining Hemmingsson’s model with data from a Danish longitudinal study
Source: BMC Public Health. 2019 Nov 7;19:1475. doi: 10.1186/s12889-019-7699-8 (PMC6839153; doi:10.1186/s12889-019-7699-8)
Supplement: Supplementary file 2 — Additional file 2: Table S2. Unadjusted estimates for the association between the individual proxies from the 5 domains (Hemmingsson:2014) and overweight and obesity at age 15, 18, 21 and 28 years (boys). [file 12889_2019_7699_MOESM2_ESM.pdf]

Table S2. Unadjusted estimates for the association between the individual proxies from the 5 domains (Hemmingsson:2014) and overweight and obesity at age 15, 18, 21 and 28 years (boys).

	**age=15** (N=1,441)		**age=18** (N=1,067)		**age=21** (N=886)		**age=28** (N=827)	
	n	OR	n	OR	n	OR	n	OR
Adult distress								
**Parental self-rated health**	1,199		857		707		621	
Good		1		1		1		1
Poor		1.5 (1.1;2.1)		1.7 (0.8;3.6)		1.2 (0.6;2.2)		0.9 (0.6;1.6)
Disharmonious family environment								
**Family functioning**	1,424		955		796		699	
Good		1		1		1		1
Poor		1.2 (0.8;1.7)		0.9 (0.4;2.1)		1.3 (0.7;2.4)		0.9 (0.5;1.5)
Offspring distress (15 years)								
**Self-esteem**	1,434		960		799		708	
High		1		1		1		1
Low		1.3 (0.9;1.9)		1.8 (0.7;4.2)		1.1 (0.5;2.3)		1.2 (0.6;2.1)
**Depressive symptoms**	1,418		954		793		700	
No		1		1		1		1
Yes		1.2 (0.9;1.7)		1.6 (0.8;3.3)		1.1 (0.6;2.1)		1.2 (0.8;1.9)
**Self-rated health**	1,435		958		793		705	
Good		1		1		1		1
Poor		2.2 (1.6;3.2)		5.1 (2.5;10.5)		2.2 (1.2;4.1)		2.4 (1.5;3.9)
Psychological and emotional overload (ages 15,18,21)								
**Avoidance coping**	1,425		954		705		497	
Low levels		1		1		1		1
High levels		1.0 (0.7;1.5)		0.8 (0.3;2.0)		0.5 (0.2;1.3)		1.6 (0.9;2.8)
**Perceived stress**	1,421		956		706		504	
Low levels		1		1		1		1
High levels		1.2 (0.8;1.7)		2.6 (1.2;5.4)		1.5 (0.8;3.0)		1.2 (0.6;2.3)
**Smoking status**	1,436		961		702		503	
Not smoking		1		1		1		1
Smoking		1.4 (0.9;2.3)		1.4 (0.5;4.1)		1.5 (0.8;2.8)		1.8 (1.1;3.0)
Homeostasis disrupted (ages 15,18,21)								
**Physical activity**	1,435		958		720		502	
High levels		1		1		1		1
Low levels		1.5 (1.1;2.1)		1.8 (0.9;3.6)		1.6 (0.8;3.1)		1.5 (0.9;2.6)
**Computer time**	1,434		959		715		501	
Low		1		1		1		1
High		1.4 (1.0;2.0)		1.2 (0.6;2.5)		1.9 (1.0;3.4)		1.6 (0.9;2.7)
